# Positive Parenting Practices and Defending Interventions in Cyberbullying: the Mediation of Disclosure

**DOI:** 10.21500/20112084.7303

**Published:** 2025-12-05

**Authors:** Agustín Morales-Álvarez, Angel Alberto Valdés-Cuervo, Lizeth Guadalupe Parra-Pérez, Ana Carolina Reyes-Rodríguez

**Affiliations:** 1 Department of Psychology, Technological Institute of Sonora. Ciudad Obregón, Sonora, México. Instituto Tecnológico de Sonora Department of Psychology Technological Institute of Sonora Ciudad Obregón Sonora Mexico; 2 Department of Education, Technological Institute of Sonora. Ciudad Obregón, Sonora, México. Instituto Tecnológico de Sonora Department of Education Technological Institute of Sonora Ciudad Obregón Sonora Mexico

**Keywords:** parenting, disclosure, bystander, cyberbullying, adolescence, crianza, revelación, espectadores, ciberacoso, adolescencia

## Abstract

Problematic adolescent Internet use has been linked to parental practices; however, the underpinnings of such relationships remain unclear. A cross-sectional study was conducted to investigate the relationship between positive parenting practices, such as warmth, inductive discipline, and autonomy support, and bystander-defending interventions in cyberbullying incidents. This study also examined the mediating role of adolescent disclosure in these relationships. The research included 684 secondary and high school students from the North of Mexico, recruited using convenience sampling. The adolescents completed self-report measures. Structural equation models with latent variables were calculated. The results showed that parental warmth had a statistically significant direct positive association with adolescents' defending interventions in cyberbullying, whereas inductive reasoning and autonomy support did not. Furthermore, the study revealed that the additive positive impact of these parenting practices had a direct effect on defender interventions in cyberbullying. Finally, the findings confirmed that adolescent disclosure played a mediating role in the relationship between all parenting practices and defensive interventions in cyberbullying. In conclusion, this research indicates that positive parenting practices encourage adolescents to intervene as defenders against cyberbullying through online disclosure.

## Resumen

El uso problemático de Internet se ha relacionado con las prácticas parentales; sin embargo, la naturaleza de estas relaciones permanece poco clara. Se llevó a cabo un estudio transversal de tipo correlacional para examinar las relaciones entre prácticas parentales positivas, tales como el afecto, la disciplina inductiva y el apoyo a la autonomía con la intervención defensiva de los espectadores en el ciberacoso. En el estudio se analizó también la mediación de la revelación en estas relaciones. La investigación incluyó a 684 estudiantes de secundaria y bachillerato del norte de México, seleccionados mediante un muestreo por conveniencia. Los adolescentes completaron medidas de autorreporte. Se calcularon modelos estructurales con variables latentes. Los resultados mostraron que el afecto parental tiene una relación directa y significativa con las intervenciones defensivas en el ciberacoso, mientras que la disciplina inductiva y el apoyo a la autonomía no se relacionaron directamente con este tipo de intervención. Adicionalmente, el estudio reveló una relación aditiva positiva directa entre estas prácticas parentales y la conducta defensiva. Finalmente, se encontró que la revelación de los adolescentes media en las relaciones entre las prácticas de crianza y las intervenciones defensivas frente al ciberacoso. En conclusión, este estudio indicó que las prácticas de crianza positivas estimulan que los adolescentes intervengan como defensores ante situaciones de ciberacoso mediante la revelación.

## 1. Introduction

Cyberbullying has become a widespread issue among adolescents worldwide (Craig et al., 2020; Zhu et al., 2021). In Mexico, according to the National Institute of Statistics and Geography (2023), 29% of adolescents are victims of cyberbullying. Cyberbullying can have a significant negative impact on adolescent development, leading to internal and external problems for those affected (Carvalho et al., 2021; Gohal et al., 2023). This phenomenon involves the intentional use of information and communication technologies (ICT) to inflict harm on an indivi dual (Olweus & Limber, 2018). Numerous studies have confirmed the critical role of those who witness or are aware of cyberbullying, known as cyber bystanders (Patrick et al., 2019; Sarmien to et al., 2019; Torgal et al., 2023).

Bystanders can actively support bullies, remain passive, defend victims, or comfort victims. Evidence indicates that when bystanders engage in defensive actions, they help mitigate the adverse effects on victims and contribute to reducing the incidence of cyberbullying (Menesini et al., 2018; Sarmiento et al., 2019). Nevertheless, despite their importance, fewer than half of the adolescents who witness cyberbullying incidents take action to defend victims. Therefore, further research is needed to understand the factors that motivate bystanders to defend victims of cyberbullying.

Previous studies have analyzed the factors that may explain adolescent defensive interventions in cyberbullying events. Most of these studies have focused on personal, peer, and situational factors (Bauman et al., 2020; Gotdiner & Gumpel, 2023). While current research highlights the significance of family characteristics in shaping adolescent prosocial behavior (Zhan & You, 2024; Wang et al., 2022), only a few studies have explored the impact of parental factors on bystander interventions in cyberbullying situations (García-Vázquez et al., 2024; Lambe et al., 2019; Machackova, 2020). These studies suggest that parental involvement and support play a crucial role in encouraging adolescents to take action to defend cyber victims.

Current research on family factors associated with bystander defending interventions in cyber- bullying is limited, with studies mainly focusing on variables such as mothers' knowledge of adolescents' online activities (DeSmet et al., 2016), expectations regarding defending interventions among parents (DeSmet et al., 2014), parental monitoring (Levy & Shela-Shayovitz, 2020), restorative discipline (García-Vázquez et al., 2024), and family support (Herry et al., 2021).

### 1.1 Positive Parenting Practices and Cyber Bystander Defending Interventions

Parenting practices involve intentional actions to achieve children's socialization goals (Yaffe, 2023). Socialization theorists posit that positive parenting practices are crucial for a child's moral development and ability to form and maintain positive peer relationships (Maccoby, 2015; Spinrad & Eisenberg, 2019). Consistent with this view, both the Motivational Model of Parenting (Skinner et al., 2005) and the parenting framework derived from Self-Determination Theory (Grolnick & Lerner, 2023) highlight a similar set of key parenting practices. Specifically, these models emphasize that parental warmth, autonomy support, and inductive discipline constitute the core of positive parenting practices.

The existing body of literature posits that these parenting practices are instrumental in fostering internalized moral values (Pastorelli et al., 2016), social skills (Han, 2021), and prosocial behaviors (Batool & Lewis, 2022; Padilla-Walker & Carlo, 2014). Parental warmth involves demonstrating empathy and support for a child's concerns and needs, expressing positive emotions, and taking positive actions during interactions (Davis & Carlo, 2018; Wang, 2019). Parents who support autonomy actively consider children's viewpoints, allow age-appropriate decision making, provide meaningful reasons for ru les, and encourage self-expression (Grolnick & Lerner, 2023; McCurdy et al., 2020). Finally, according to some studies (Guevara et al., 2015; Lansford, 2019), inductive discipline enables parents to encourage children to con- sider the consequences of their misbehavior on others' well-being. Previous studies have confirmed a link between parenting practices and online prosocial behaviors in adolescents (Padilla-Walker et al., 2020; Wang et al., 2022). However, these studies have not exa- mined the role and the mechanism of these practices in bystander defender interventions in cyberbullying among adolescents.

### 1.2 The Mediating Role of Adolescent Disclosure

The role of adolescent agency is emphasized in the conceptual model proposed by Soenens and Vansteenkiste (2020). The model under- scores the role of adolescent agency in determining how parental practices affect adolescents. The model proposes that adolescents' responses to parental practices serve as a critical mechanism for explaining the effect of parental behaviors on adolescents' outcomes. Adolescents' responses to parental behaviors involve managing information about unsupervised activities with their parents. They may choose to share or withhold information with their parents based on an appraisal of their parents' behaviors (Elsharnouby & Dost-Gozkan, 2020; Padilla-Walker et al., 2020). Adolescent disclosure with parents about their online activities, whereabouts, and relationships is the primary source of parents' knowledge about their adolescents' online lives (Cutrín et al., 2019; Lionetti et al., 2016). The need for more autonomy and privacy du- ring adolescence, the time adolescents spend on media, and the limited access parents have to information regarding adolescents' media use explain why adolescent disclosure is necessary for parents to know about their adolescents' media-related activities.

Studies examining the link between specific parental practices and adolescents' disclosure of their media activities to parents have concluded that positive parenting practices encourage adolescents to share information about their activities with their parents (Padilla-Walker et al., 2020; Son & Smetana, 2024). Such findings have led some scholars (Padilla-Walker & Son, 2019; Wang, 2019) to posit that these practices facilitate disclosure by promoting favorable parent-adolescent relations and the internalization of values.

Empirical research has shown that adolescent disclosure of media-related activities is an influential protective factor against risky online behaviors, social interactions with strangers (Dotterer & Day, 2019; Martin et al., 2020), and online privacy concerns (Shin & Kang, 2016). Additionally, it is associated with positive outcomes, such as higher levels of prosocial behavior (Laible et al., 2019; Padilla-Walker et al., 2020) and skills to handle online problems, such as cyberbullying (Levy & Sela-Shayovitz, 2020; Rinaldi et al., 2023). The literature review revealed a significant gap in empirical research on the relationship between media disclosure and adolescents' defender interventions in cyberbullying situations.

### 1.3 The Present Study

**Figure 1:**
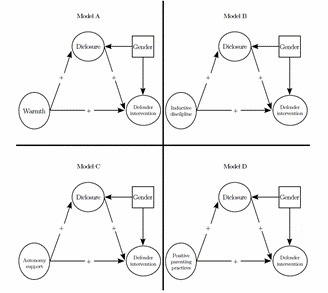
Hypothesized Structural Models for Unique and Additive Parenting Practices Associations with Adolescents' Disclosure and De- fending Intervention in Cyberbullying

Several studies have suggested that parental practices are associated with adolescent online behaviors through disclosure. Drawing on models of positive parenting, we proposed three models in which parental warmth (Mo- del A), autonomy support (Model B), and inductive discipline (Model C) each have a unique direct and indirect effect on adolescents' defender interventions against cyber-bullying via adolescent disclosure. Additionally, we tested the direct and indirect additive effects of such parental behaviors (Model D) on bystander defender interventions in cyber-bullying via adolescents' disclosure. Given that the research reported gender differences in bystander-defending interventions in cyberbullying (Macaulay et al., 2019; Wang & Kim, 2024), we statistically controlled for gender in our study (see Figure 1).

The following hypotheses were proposed to achieve this research: Hypothesis 1 (unique direct and mediating relationships of parental practices): We expected that parental warmth, inductive reasoning, and autonomy granting would each positively predict adolescents' bystander defense behaviors in response to cyberbullying events. We also expected that adolescents' self-disclosure would partially mediate these relationships.

Hypothesis 2 (additive direct and mediating relationship): We further hypothesized that the additive effect of these parental practices would predict adolescents' bystander defending behaviors during cyberbullying episodes. Additionally, we expected that adolescents' media disclosure would partially mediate this additive relationship.

## 2. Method

### 2.1 Participants

The study employed a cross-sectional design, gathering data through non-probabilistic convenience sampling from 12 public secondary and 12 public high schools in So nora and Tabasco, Mexico. Public schools in Mexico serve the largest student populations at these educational levels, representing a range of socioeconomic backgrounds, predominantly from low- and middle-income households (Graña & Murillo, 2023).

Questionnaires were administered to a prearranged group of students at each school, with the principals' approval. The inclusion criteria were as follows: (1) parents were required to return a signed informed consent form; (2) students needed to provide their written assent to participate voluntarily; and (3) participants must live with at least one parent 50% of the time. Ultimately, 684 valid questionnaires were obtained, with 56% of respondents being female. The students' ages ranged from 11 to 18 years, with a mean of 14.3 years ( *SD* = 1.75).

### 2.2 Measures

#### Positive Parental Practices

Drawing on the work conducted by several scholars (Davis & Carlo, 2018; Mageau et al., 2015), we developed a new scale to assess domain-specific parenting practices that influence adolescents' Internet use. The scale measures: parental warmth (5 items, e.g., “My parents are interested in my needs regarding Internet use”, “My parents are aware of my Internet activities”), parental inductive discipline (7 items, e.g., “My parents talk to me about the importance of following the ru les they set about the Internet”, “My parents explain to me that there are consequences for my behavior on the Internet”), and parental autonomy support (5 items, “My parents allow me to choose my activities and how I spend my time online, within certain limits”, “My parents give me many opportunities to make my own decisions about what I do online”). These scales were rated on a five-point Likert scale (0 = never to 4 = always).

#### Disclosure

We used items from scales reported in previous research to assess how often adolescents disclose their online activities, whereabouts, and relationships to their parents (Lionetti et al., 2016; Padilla-Walker et al., 2020). The scale comprises six items (e.g., “I share with my parents about the people that are part of my digital communities (forums, WhatsApp, social sites)”, “I talk to my parents about my online friends”). The items were rated on a five-point Likert scale (0 = never to 4 = always).

#### Defending Intervention in Cyberbullying

This study used the defending intervention subscale of the Cyberbullying Bystander Scale (Sarmiento et al., 2019), encompassing indicators of both online interventions. The subscale includes four items that belong to online intervention (e.g., "When I am on social media, and I see some people harass others who cannot defend themselves," "I tell them not to do this") and seven items related to the offline intervention (e.g., "If I know that someone publishes gossip or rumors about others on the Internet," "I tell them in person to stop"). Participants responded to the items using a 5-point Likert-type scale (0 = never to 4 = always).

### 2.3 Ethical Considerations and Procedure

The university's ethics committee approved the research protocol (official letter no. 168), confirming that the study adhered to the ethical principles outlined in the American Psychological Association (APA) guidelines. After receiving approval, the authors approached the school principals and teachers for their cooperation. The principal invited the parents to sign a consent letter authorizing their children's participation in the study. Only 12% of parents refused to consent to their children participating. The adolescents were informed about the study's purpose, ensured that their responses would remain confidential, and were informed that they could withdraw from the study at any time. All adolescents signed an assent form to respond to the questionnaires. The scales measuring the input va riables (parental practices) and the output variables (disclosure and defending intervention) were administered in two separate classroom sessions, with a one-month gap between them to reduce common method variance (Podsakoff et al., 2003).

### 2.4 Data Analysis

#### Software

Preliminary data screening, which included analyses of missing values, examination of univariate distributions, and assessment of common-method variance, was conducted prior to further analyses. Subsequently, descriptive and correlational analyses were performed, followed by the application of structural equation modeling (SEM). All statistical analyses were conducted utilizing JASP version 0.19.

#### Preliminary Data Analyses

The variables with missing data (less than 5%) were handled using the Full Information Maximum Likelihood method. To assess the normality of item score distributions, both skewness and kurtosis were examined. In accordance with the established guidelines in the field of so cial science, a threshold of ±2 was used as an initial criterion to identify potential departure from normality (Bandalos, 2018; George & Mallery, 2019).

Since the study relied on adolescents' self-reported responses, we also evaluated whether common method variance (CMV) might have introduced bias into the results. To do this, we used the unmeasured latent method, comparing a model that includes all measured variables to an alternative model incorporating a latent construct linked to all observable indicators. The absence of statistically significant differences between the two models was considered evidence of no CMV bias (Baumgartner & Weijters, 2021).

#### Psychometric Analyses

Confirmatory factor analyses (CFAs) were conducted to examine the scales' internal structure. The models were estimated with the Robust Maximum Likelihood estimator (MLM), incorporating Satorra and Bentler's corrected/scaled chi-square statistics (S- B X^2^). Model fit was evaluated using the following criteria: S-Bx2 value *p* > .001; CFI and TLI > .95; SRMR and RMSEA < .08 (Bandalos, 2018; Kline, 2023). Scale reliability was evaluated using McDonald's omega coefficient (w), with valúes > .70 considered acceptable (Bandalos, 2018).

#### Descriptive and Correlational Analyses

Descriptive analysis was performed, including the calculation of mean and standard deviation. Moreover, we asses- sed bivariate correlations using Spear- man's coefficient. According to the literature, the small, medium, and large effects were determined to be *r* = .10, *r* = .20, and *r* = .30, respectively (Funder & Ozer, 2019; Gignac & Szodorai, 2016).

#### Structural Equation Modeling

Multiple structural equation models were conducted. To address the issue of multivariate non-normality, we utilized the Robust Maximum Likelihood Estimation recommended by Mueller and H ancock (2019). The study employed several goodness-of-fit indices, including the Satorra-Bentler Chi-square statistic with their associated probability (S-BX2 with *p* > .001), Comparative Fit Index (CFI > .95), Tucker-Lewis Index (TLI > .95), Standardized Root Mean Square Residual (SRMR < .08), and Root Mean Square Error of Approximation (RMSEA < .08) with a 90% confidence interval, to evaluate model fit (Kline, 2023).

## 3. Results

### 3.1 Preliminary Analyses

All scale items showed skewness and kurtosis values within acceptable limits (±2; Bandalos, 2018; George & Mallery, 2019), indicating no substantial departures from normality. For the CMV analysis, the comparison between the two models revealed no statistically significant difference (Ax2 = 3.82, *df* = 1, *p* = .051). Consistent with Baumgartner and Weijters (2021), this result indicates that CMV bias was not a significant concern in our study (Baumgartner & Weijters, 2021).

### 3.2 Psychometric Analyses

To obtain evidence of validity concerning the internal structure of the measures used in the study, confirmatory factor analyses (CFAs) were performed. A summary of the findings is provided in Table 1. The results revealed that the proposed measurement models demonstrated a satisfactory fit to the data, with CFI and TLI values > .95 and SRMR and RMSEA < .08. All standardized coefficients were statistically significant (*p* < .001).

McDonald's omega coefficients indicated that internal consistency for the positive parenting practices (warmth: w = .94; inductive discipli ne: w = .84; autonomy support: w = .97), disclosure (w = .89), and defending intervention in cyberbullying (w = .91), suggesting that the corresponding scores demonstrated satisfactory reliability.

**Table 1:** Summary of Fit Indices for the Scales In volved in the Study

Measure	S-BX^2^	*df*	SRMR	CFI	TLI	RMSEA [90% CI]
Positive parental practices	654.74***	202	.04	.95	.95	.05 [.06, .07]
Disclosure	15.66*	7	.01	.99	.99	.05 [.02, .08]
Defending intervention in cyberbullying	71.52***	39	.02	.99	.99	.04 [.02, .06]

*p < .05. ***p < .001.

### 3.3 Descriptive and Correlational Analyses

Table 2 indicates that adolescents perceived their parents as rarely providing warmth and as sometimes using inductive discipline to manage adolescents' media-related misbehavior, but rarely using autonomy-supportive practices. Furthermore, the study found that adolescents seldom shared their Internet activities with their parents. Moreover, when cyberbullying incidents occurred, most adolescents reported that they seldom intervened to defend victims against aggressors. These results show a moderate tendency among students to share information with parents about their online activities, relationships, and whereabouts. In contrast, defending behavior in cyberbullying events was reported to be infrequent.

Spearman's correlations showed that these parental practices were positively associated with adolescents' disclosure and bystander defending interventions. Being female was positively correlated with disclosure and defender intervention in cyberbullying. The effect sizes of the statistically significant correlations among study variables ranged from large to moderate, suggesting theoretical and practical implications for these associations.

**Table 2:** Descriptive Statistics and Correlations Between Variables Involved in the Study

Variable	*M*	*SD*	1	2	3	4	5	6
1. Parental warmth	2.56	1.05	_-_					
2. Parental discipline inductive	3.04	1.00	.69***	-				
3. Parental support autonomy	2.70	1.04	.64***	.61***	-			
4. Adolescent disclosure	2.60	1.19	.68***	.65***	.50***	^-^		
5. Bystander intervention defending	1.63	1.21	.23***	.19***	.18***	.25***	-	
6. Gender			.01	.08	.02	.15***	.16***	-

*Note*. Adolescents' gender was coded as 0 = Male, 1 = Female.

***p < .001.

### 3.4 Structural Equation Modeling

The findings from the unique effect models indicate an adequate fit to the data (see Table 3). In terms of direct relationships, three positive parental practices-warmth, inductive discipline, and autonomy support- were significantly associated with adolescents' disclosure (3 = .80, *p <* .001; 3 = .68, *p <* .001; 3 = .60, *p* < .001, respectively). However, only parental warmth showed a significant direct association with defending behavior in cyberbullying situations (3 = .18, *p* = .042). Across the three models, adolescents' disclosure emerged as a significant mediator linking each parenting practice to bystander defending responses (Model A: 3 = .11, *p* = .011; Model B: 3 = .14, *p* = .003; Model C: 3 = .13, *p* = .002).

The results regarding the additive effect of positive parental practices indicated that the model incorporating the additive effect of these practices fit the data (see Table 3). Positive parenting practices were significantly and positively associated with defending in cyberbullying events (3 = .14, *p* = .039). Furthermore, the study revealed a statistically significant indirect link between positive parental practices and defending interventions (3 = .13, *p* = .009), mediated by adolescents' disclosure of their online activities and whereabouts. Table 4 summarizes the direct and indirect effects for both unique and additive models.

Overall, the findings indicate that these positive parenting practices significantly influence how adolescents respond when they witness cyberbullying. While only parental warmth exhibited a direct association with defending behavior in the individual models, all three practices were linked to adolescents' disclosure of their online experiences. This disclosure served as a key mechanism connecting each parenting practice to adolescents' defense intervention in cyberbullying. Notably, when these parenting practices were considered together in the additive model, their combined influence did show a direct relationship with more frequent defending behaviors.

**Table 3:** Summary of Fit Indices of Mediation Models

Model	S-Bχ2	*df*	SRMR	CFI	TLI	RMSEA	[90% CI]
Model A (Warmth)	435.49***	181	.04	.95	.95	.060	[.053, .068]
Model B (Inductive discipline)	603.82***	291	.04	.95	.95	.055	[.049, .062]
Model C (Autonomy support)	424.91***	180	.05	.96	.96	.059	[.052, .067]
Model D (Additive model)	964.84***	509	.05	.95	.95	.061	[.045, .075]

***p < .001.

**Table 4:** Standardized Direct and Indirect Effects, and Explained Variance (R²) for the Unique and Additive Models

Effects	Model A (Warmth)	Model B (Inductive discipline)	Model C (Autonomy support)	Model D (Additive effects)
Direct effect				
Predictor**→**Disclosure	.80*** (.74, .85)	.68*** (.54, .78)	.60*** (.58, .72)	.71*** (.69, .80)
Predictor→Defender	.18* (.09, .27)	.10^n/s^ (-.02, .19)	.11n/s (-.02, .22)	.14* (.08, .21)
Disclosure→Defender	.13* (.04, .22)	.21** (.09, .24)	.21** (.12, .24)	.19** (.07, .23)
Gender→Disclosure (control variable)	.05^n/s^ (-.04, .26)	.02^n/s^ (-.01, .13)	.05^n/s^ (-.07, .32)	.02^n/s^ (-.12, .22)
Gender→Defender (control variable)	.16*** (.11, .30)	.15*** (.10, .26)	.16*** (.11, .29)	.16*** (.10, .29)
Indirect effect				
Predictor→Disclosure→ Defender	.11* (.05, .19)	.14** (.06, .21)	.13** (.06, .18)	.13** (.06, .18)
Explained variance (R^2^)				
R^2^ Defender intervention	.12	.11	.11	.15

*Note.* Standardized coefficients are reported. 95% confidence intervals are presented in parentheses. Gender was a control variable; 0 = Male, 1 = Female. n/s = non-significant relationship.

*p < .05. **p < .01. ***p < .001.

## 4. Discussion

Previous research suggests that positive parenting can foster prosocial behavior among adolescents in online environments. However, limited information is available on how parenting practices affect cyber bystander behaviors and the factors that may mediate these relationships. To address this gap, the parental socialization framework was used to explore the direct and indirect links between positive parenting practices, disclosure, and bystander-defending actions in cyberbullying situations. While not all the hypotheses were supported, the results indicated that positive parenting can encourage adolescents to intervene when they witness cyberbullying. Additionally, the analysis revealed that adolescents' openness about their online activities, whereabouts, and relationships plays a mediating role in the link between positive parenting and intervening when they see cyberbullying.

### 4.1 Unique and Additive Direct Relationship of Positive Parenting Practices

This study examined the link between pa- rental warmth and adolescents' responses to cyberbullying. Our findings showed a direct relationship between warmer parenting and adolescent intervention as defenders in cyberbullying situations. This result aligns with previous studies that posited an association between parental warmth and proso cial behaviors in adolescents (Pastorelli et al., 2016; Putnick et al., 2018). According to Pastorelli et al. (2021), warm parenting fulfills children's needs for trust and protection while fostering empathetic, other-oriented behaviors that adolescents may emulate with peers. Moreover, this parenting practice may enable adolescents to internalize their parents' behavior as a benchmark for evaluating their actions in diverse social situations (Xu & Zheng, 2023; Buckley et al., 2024).

The study failed to establish a direct link between inductive parental discipline and autonomy support with bystander-defending interventions. However, previous research suggests that parenting practices grounded in reasoning and moral reflection tend to foster prosocial outcomes in adolescents (Padilla-Walker et al., 2020; Wang et al., 2022), while restorative discipline is not conceptually explained or reasoned. Additional studies are needed to explain this divergence. Nevertheless, it may be attributable to certain variables that moderate these relationships, such as the cost of defending interventions, anonymity of responses, and peer behaviors in cyberbullying incidents.

The study provides evidence supporting the hypothesis of a direct additive positive link between positive parenting practices and bystander-defending interventions. This result is noteworthy because previous research has typically examined these practices separately, without considering their interdependence. Essentially, the combination of parental warmth, autonomy support, and inductive discipline influenced the bystander defender's intervention in cyberbullying. Although further research is required, the findings of the present study suggest that parental warmth enhances the effects of autonomy support and inductive discipline in defending interventions against cyberbullying.

### 4.2 Mediating Role of Disclosure

Research indicates that how adolescents manage information plays a critical role in determining the influence of parenting practices on their development (Padilla-Walker et al., 2020; Song & Smetana, 2024). The current study extends these findings by examining the effect on cyber bystander behavior. We found that adolescent disclosure mediates the link between parental warmth, inductive dis cipline, and autonomy support with bystander defender interventions in cyberbullying. These findings suggest that adolescent disclosure is a critical factor in explaining why positive parenting practices are associated with defender interventions in cyberbullying. This result confirms the value of adolescents' agency, especially how they respond to their parents' behaviors, in understanding the effect of parenting practices on their development (Soenens & Vansteenkiste, 2020).

### 4.3 Theoretical and Practical Implications

According to the theoretical model of parenting (Grolnick & Lerner, 2023; Skinner et al., 2005) and empirical evidence (Davis & Carlo, 2018; van der Storm et al., 2022), this study confirmed that the parental practices examined are associated with developmental outcomes in adolescents. These parenting practices promote prosocial online behaviors among adolescents towards their peers. Although further studies are necessary, the results suggest that warmth is essential for parents to exert an effect on defender interventions. The findings also confirm the use- fulness of the parental knowledge framework in explaining adolescent behavior in online settings, as proposed by some scholars (Martin et al., 2020; Dotterer & Day, 2019), and the link between adolescent disclosure and defen der intervention in cyberbullying. Furthermore, the findings also highlighted the value of considering adolescent agency, especially how adolescents respond to parental practices, to understand the different influences of parents on adolescent development outcomes (Maccoby, 2015; Soenens & Vansteenkiste, 2020). Effective cyberbullying prevention requires educating parents on positive parenting practices. Moreover, parents must encourage open communication with their adolescents regarding online safety and responsible behaviors in online settings. Particularly important is that research suggests that parents positively impact adolescents' development when they engage in socialization efforts that lead to constructive responses from adolescents, such as disclosure about their activities and relationships.

### 4.4 Limitations

Although this study yielded significant findings, it is essential to consider its limitations. The sample was collected from urban areas of Mexico, which may limit the generalizability of the findings to other populations, such as indigenous or rural adolescents, owing to the country's diverse cultural makeup. Future research should include diverse demographic groups, such as adolescents from different ethnicities, socioeconomic backgrounds, and rural areas. Additionally, the study relied on self-reported data from adolescents, which may have been biased. Other measures, such as observation and input from multiple sour ces, including parents, should be employed to address this limitation. It also considers the study's non-experimental mediation design, which limits our ability to establish cau sal relationships among the study variables or to observe their temporal relationships. Different mediators and models may fit the data. Therefore, future research should explo re other mediators and use longitudinal or experimental data to better understand the links between the study variables. Finally, although previous empirical research has indicated that the effect of parenting practices on adolescents' social behavior may vary across parent-child dyads (e.g., mother-son, mother-daughter, father-son, and father-daughter), our study did not differentiate between mothers' and fathers' reports on adolescent measures. This limitation highlights the need for future research to explore whether the parenting practices evaluated in this study function similarly or differently across specific parent-adolescent dyads.

## 5. Conclusions

Adolescents spend more time in online settings, and parents can mediate and moni tor adolescents' online activities effectively or ineffectively. Research has confirmed that when parents focus on providing the- se practices, adolescents are more likely to disclose their media-related activities and experiences. This information sharing, in turn, is linked to adolescent interventions in cyberbullying. These findings suggest that positive parenting practices can promote prosocial online behaviors in adolescents. Parents need to offer warmth while encouraging adolescents to play an active role in managing their media use and looking out for the well-being of others online.

Researchers should examine additional variables associated with adolescents' agency, such as their appraisal of parental practices. This appraisal should mediate the relationship between parenting practices and defender interventions in cyberbullying events. Further, researchers should explore how the effects of positive parenting practices on disclosure and advocacy interventions vary between mothers and fathers.

## References

[B1] Bandalos D. L (2018). Measurement theory and applications for the social sciences.

[B2] Batool S. S., Lewis C. A (2022). Does positive parenting predict pro social behavior and frienship quality among adolescents? Emotional intelligence as a mediatior. Current Psychology.

[B3] Bauman S., Yoon J., Iurino C., Hackett L (2020). Experiences of adolescent witnesses to peer victimization: The bystander effect. Journal of School Psychology.

[B4] Baumgartner H., Weijters B (2021). Dealing with common method variance in international marketing research. Journal of International Marketing.

[B5] Buckley L., Atkins T., Perera W., Waller M (2024). Trajectories of parental warmth and the role play in explaining adolescent prosocial behavior. Journal of Youth and Adolescence.

[B6] Carvalho M., Branquinho C., Gaspar de Matos M (2021). Cyberbullying and bullying: Impact on psychological symptoms and well-being. Child Indicators Research.

[B7] Craig W., Boniel-Nissin M., King N., Walsh S. D., Boer M., Donnelly P. D., Harel-Fisch Y., Malinowska-Cieslik M., Gaspar de Matos M., Cosma A., Van den Eijnden R., Vieno A., Elgar F. J., Molcho M., Bjereld Y., Pickett W (2020). Social media use and cyber-bullying: A cross-national analysis of young people in 42 countries. Journal of Adolescent Health.

[B8] Cutrín O., Maneiro L., Sobral J., Gómez-Fraguela J. A (2019). Longitudinal validation of a new measure to assess parental knowledge and its sources in Spanish adolescents. Journal of Child and Family Studies.

[B9] Davis A. N., Carlo G (2018). The roles of parenting practices, sociocognitive/ emotive traits, and prosocial behaviors in low-income adolescents. Journal of Adolescence.

[B10] DeSmet A., Bastiaensens S., Van Cleemput K., Poels K., Vandebosch H., Cardon G., De Bourdeaudhuij I (2016). Deciding whether to look after them, to like it, or leave it: A multidimensional analysis of predictors of positive and negative bystander behavior in cyberbullying among adolescents. Computers in Human Behavior.

[B11] DeSmet A., Veldeman C., Poels K., Bastiaensens S., Van Cleemput K., Vandebosch H, De Bourdeaudhuij I (2014). Determinants of self-reported bystander behavior in cyberbullying incidents amongst adolescents. Cyberpsychology, Behavior, and Social Networking.

[B12] Dotterer A. M., Day E (2019). Parental knowledge discrepancies: Examining the roles of warmth and self-disclosure. Journal of Youth and Adolescence.

[B13] Elsharnouby E., Dost-Gozkan A (2020). Adolescents' well-being with respect to the patterns of disclosure to and secrecy from parents and the best friend: A person- centered examination. Journal of Youth and Adolescence.

[B14] Funder D. C., Ozer D. J (2019). Evaluating effect size in psychological research: Sense and nonsense. Advances in Methods and Practices in Psychological Science.

[B15] García-Vázquez F. I., Valdés-Cuervo A. A., León-Parada M. D., Parra-Pérez L. G (2024). Restorative parental discipline and types of defending bystander intervention in cyberbullying: The mediate role of justice sensitivity. Cyberpsychology, Behaviors, and Social Networking.

[B16] George D., Mallery P (2019). IBM SPSS Statistic 26 step by step. A simple guide and reference.

[B17] Gignac G. E., Szodorai E. T (2016). Effect size guidelines for individual differences researchers. Personality and Individual Differences.

[B18] Gohal G., Alqassim A., Elyeb E., Rayyani A., Hakami B., Al Faqih A., Hakami A., Qadri A., Mahfouz M (2023). Prevalence and related risk of cyberbullying and its effects on adolescent. BMC Psychiatry, 23.

[B19] Gotdiner V., Gumpel T. P (2023). Bystander intervention style and motivational factors influencing behavior in bullying situations. Psychology in the Schools.

[B20] Graña R., Murillo F. J (2023). Una mirada a la segregación escolar por nivel socioeconómico en México y sus entidades federativas [A look at school segregation by socioeconomic level in Mexico and its federal entities].. Revista Mexicana de Investigación Educativa.

[B21] Grolnick W. S., Lerner R. E, Ryan R. M. (2023). The Oxford handbook of self-determination theory.

[B22] Guevara I. P., Cabrera V. E., Gonzalez M. R., Devis J. V (2015). Empathy and sympathy as mediators between parental inductive discipline and prosocial behaviors in Colombian families. International Journal of Psychological Research.

[B23] Han S (2021). Reproducing the working class? Incongruence between the valuation of social-emotional skills in school and in the labor market. Sociological Perspectives.

[B24] Herry E., Gonültas S., Mulvey K. L (2021). Digital era bullying: An examination of adolescent judgments about bystander intervention online. Journal of Applied Developmental Psychology.

[B25] Kline R. B (2023). Principles and practice of structural equation modeling.

[B26] Laible D., Conover O., Lewis M. E., Karahuta E., Norden C. V., Stout W., Carlo G., Cruz A (2019). The quality of mother-adolescent disclosure: Links with predictors and adolescents' sociomoral outcomes. Social Development.

[B27] Lambe L. J., Cioppa V. D., Hong I. K., Craig W. M (2019). Standing up to bullying: A social ecological review of peer defending in offline and online contexts. Aggression and Violent Behavior.

[B28] Lansford J. E, Laible D. J., Carlo G., Padilla-Walker L. M. (2019). The Oxford handbook of parenting and moral development.

[B29] Levy M., Sela-Shayovitz R (2020). Cyberaggression: The effect of parental monitoring on bystander roles. International Journal of Child, Youth & Family Studies.

[B30] Lionetti F., Keijsers L., Dellagiulia A., Pastore M (2016). Evidence of factorial validity of parental knowledge, control and solicitation, and adolescent disclosure scales: When the ordered nature of Likert scales matters. Frontiers in Psychology.

[B31] Machackova H (2020). Bystander reactions to cyberbullying and cyberaggression: Individual, contextual, and social factors. Current Opinion in Psychology.

[B32] Macaulay P. J. R., Boulton M. J., Betts L. R (2019). Comparing early adolescents' positive bystander responses to cyberbullying and traditional bullying: The impact of severity and gender. Journal of Technology in Behavioral Science.

[B33] Maccoby E. E, Grusec J., Hastings P. D. (2015). Handbook of socialization: Theory and research.

[B34] Mageau G. A., Ranger F., Joussemet M., Koestner R., Moreau E., Forest J (2015). Validation of the Perceived Parental Autonomy Support Scale (P-PASS). Canadian Journal of Behavioural Science.

[B35] Martin F., Hunt B., Wang C., Brooks E (2020). Middle school student perception of technology use and digital citizenship practices. Computers in the Schools.

[B36] McCurdy A. L., Williams K. N., Lee G. Y., Benito-Gomez M., Fletcher A. C (2020). Measurement of parental autonomy support: A review of theoretical concerns and developmental considerations. Journal of Family Theory & Review.

[B37] Menesini E., Zambuto V., Palladino B. E, Campbell M., Bauman S. (2018). Reducing cyberbul lying in schools: International evidence-based best practices.

[B38] Mueller R. O., Hancock G. R, Hancock G. R., Stapleton L. M., Mueller R. O. (2019). The reviewer's guide to quantitative methods in the social sciences.

[B39] National Institute of Statistics and Geography (2023). Módulo sobre el ciberacoso.

[B40] Olweus D., Limber S. P (2018). Some problems with cyberbullying research. Current Opinion in Psychology.

[B41] Padilla-Walker L. M., Carlo G, Padilla-Walker L. M., Carlo G. (2014). Prosocial development: A multi- dimensional approach.

[B42] Padilla-Walker L. M., Son D (2019). Longitudinal associations among routine disclosure, the parent-child relationship, and adolescents' prosocial and delinquent behaviors. Journal of Social and Personal Relationships.

[B43] Padilla-Walker L. M., Stockdale L. A., Son D., Coyne S. M., Stinnett S. C (2020). Associations between parental media monitoring style, information management, and prosocial and aggressive behaviors. Journal of Social Personal Relationships.

[B44] Pastorelli C., Landsford J. E., Kanacri B. P. L., Malone P. S., Di Giunta L., Bachinni D., Bombi A. S., Zelli A., Miranda M. C., Bornstein M. H., Tapanya S., Uribe L. M., Alampay L. P., Al-Hassan S. M., Chang L., Deater-Deckard K., Dodge K. A., Oburu P., Skinner A. T., Sorbring E (2016). Positive parenting and children's prosocial behavior in eight countries. The Journal of Child Psychology and Psychiatry.

[B45] Pastorelli C., Zuffiano A., Lansford J. E., Thartori E., Bornstein M. H., Chang L., Deater-Deckard K., Di Giunta L., Dodge K. A., Gurdal S., Liu Q., Long Q., Oburu P., Skinner A. T., Sorbring E., Steinberg L., Tapanya S., Uribe Tirado L.M., Bachinni D (2021). Positive youth development: Parental warmth, values, and prosocial behavior in 11 cultural groups. Journal of Youth Development.

[B46] Patrick R. B., Rote W. M., Gibbs J. C., Basinger K. S (2019). Defend, stand by, or join in?: The relative influence of moral identity, moral judgment, and social self-efficacy on adolescents' bystander behaviors in bullying situations. Journal of Youth and Adolescence.

[B47] Podsakoff P. M., MacKenzie S. B., Lee J.-Y., & Podsakoff N. P (2003). Common method biases in behavioral research: A critical review of the literature and recommended remedies. Journal of Applied Psychology.

[B48] Putnick D. L., Bornstein M. H., Lansford J. E., Chang L., Deater-Deckard K., Di Giunta L., Dodge K. A., Malone P. S., Oburu P., Pastorelli C., Skinner A. T., Sorbring E., Tapanya S., Uribe Tirado L. M., Zelli A., Peña Alampay L., Al-Hassan S. M., Bacchini D., Bombi A. S (2018). Parental acceptance-rejection and child prosocial behavior: Developmental transactions across the transition to adolescence in nine countries, mothers and fathers, and girls and boys. Development Psychology.

[B49] Rinaldi C. M., Bulut O., Muth R., Di Stasio M (2023). The influence of parenting dimensions and junior high school students' involvement in bullying. Journal of School Violence.

[B50] Sarmiento A., Herrera-López M., Zych I (2019). Is cyberbullying a group process? Online and offline bystanders of cyberbullying act as defenders, reinforces and outsiders. Computers in Human Behavior.

[B51] Shin W., Kang H (2016). Adolescents' privacy concerns and information disclosure online: The role of parents and the Internet. Computers in Human Behavior.

[B52] Skinner E., Johnson S., Snyder T (2005). Six dimensions of parenting: A mtivational model. Parenting: Science and Practice.

[B53] Soenens B., Vansteenkiste M (2020). Taking adolescents' agency in socialization seriously: The role of appraisals and cognitive- behavioral responses in autonomy-relevant parenting. New Directions for Child and Adolescent Development.

[B54] Song G., Smetana J. G (2024). Longitudinal associations among psychological control, positive and negative interactions, and adolescents' domain-specific disclosure to parents. Journal of Youth and Adolescence.

[B55] Spinrad T. L., Eisenberg N, Laible D. J., Carlo G., Padilla Walker L. M. (2019). The Oxford handbook of socialization.

[B56] Torgal C., Espegale D. L., Polanin J. R., Ingram K. M., Robinson L. E., El Sheikh A. J., Valido A (2023). A meta-analysis of school- based cyberbullying prevention programs' impact on cyber-bystander behavior. School Psychology Review.

[B57] Van der Storm L., Van Lissa C. J., Lucassen N., Helmerhorst K. O. W., Keizer R (2022). Maternal and paternal parenting and child prosocial behavior: A meta-analysis using a structural equation modeling design. Marriage & Family Review.

[B58] Wang M (2019). Harsh parenting and adolescent aggression: Adolescents' effortful control as the mediator and parental warmth as the moderator. Child Abuse & Neglect.

[B59] Wang H., Geng J., Liu K., Wei X., Wang J., Lei L (2022). Future time perspective and self-control mediate the links between parental autonomy support and adolescents' digital citizenship behavior. Youth & Society.

[B60] Wang S., Kim K. J (2024). Effects of victimization experience, gender, and empathic distress on bystanders' intervening behavior in cyberbullying. The Social Science Journal.

[B61] Xu J., Zheng Y (2023). Parent- and child-driven daily family stress processes between daily stress, parental warmth, and adolescent adjustment. Journal of Youth and Adolescence.

[B62] Yaffe Y (2023). Systematic review of the differences between mothers and fathers in parenting styles and practices. Current Psychology.

[B63] Zhan W., & You Z (2024). Family communication patterns, self- efficacy, and adolescent online prosocial behavior: a moderated mediation model. Humanities and Social Science Communication.

[B64] Zhu C., Huang S., Evans R., Zhang W (2021). Cyberbullying among adolescents and children: A comprehensive review of the global situation, risk factors, and preventive measure. Frontiers in Public Health.

